# Charge-state resolved laser acceleration of gold ions to beyond 7 MeV/u

**DOI:** 10.1038/s41598-022-08556-8

**Published:** 2022-03-21

**Authors:** F. H. Lindner, E. G. Fitzpatrick, D. Haffa, L. Ponnath, A.-K. Schmidt, M. Speicher, B. Zielbauer, J. Schreiber, P. G. Thirolf

**Affiliations:** 1 Ludwig-Maximilians-Universität München, Am Coulombwall 1, 85748 Garching bei München, Germany; 2 GSI Helmholtzzentrum für Schwerionenforschung GmbH, Planckstraße 1, 64291 Darmstadt, Germany

**Keywords:** Astronomy and planetary science, Optics and photonics, Physics

## Abstract

In the past years, the interest in the laser-driven acceleration of heavy ions in the mass range of $\text {A}\approx 200$ has been increasing due to promising application ideas like the fission-fusion nuclear reaction mechanism, aiming at the production of neutron-rich isotopes relevant for the astrophysical *r*-process nucleosynthesis. In this paper, we report on the laser acceleration of gold ions to beyond 7 MeV/u, exceeding for the first time an important prerequisite for this nuclear reaction scheme. Moreover, the gold ion charge states have been detected with an unprecedented resolution, which enables the separation of individual charge states up to 4 MeV/u. The recorded charge-state distributions show a remarkable dependency on the target foil thickness and differ from simulations, lacking a straight-forward explanation by the established ionization models.

## Introduction

Promising application perspectives for laser-accelerated heavy ions in the mass range of $\text {A}\approx 200$ led to an awakening interest in laser-based heavy ion acceleration. Since 2015, multiple experimental papers reported on progress in laser-driven acceleration of gold ions, pushing the achieved kinetic energies from 1 MeV/u^1^ to 5 MeV/u^2^ to finally 6.1 MeV/u^3^. This evolution has been accompanied by several simulations^4–6^, which especially studied the expected gold ion charge-state distributions based on the established models of tunnel and electron impact ionization.

With this paper, we pursue the long-term goal of realizing the fission-fusion reaction mechanism proposed already a decade ago^7^. This aims at the production of extremely neutron-rich isotopes close to the waiting point of the rapid neutron capture (*r*-)process at the magic neutron number $\text {N}=126$^8^, which is a decisive region for the astrophysical nucleosynthesis of the heaviest elements in the Universe. The fission-fusion reaction mechanism is a two-step process, which is expected to be enabled to occur when ultra-dense bunches of laser-accelerated heavy, fissile ions (like $^{232}$Th) with kinetic energies above the fission barrier impinge on a target consisting of the same material. In a first step, both projectile and target ions undergo fission. Afterwards, fusion of fission fragments may happen, in case of fusion between two light fission fragments the desired neutron-rich *r*-process isotopes are formed. This reaction scheme requires the application of laser-accelerated heavy ion bunches owing to their ultra-high, almost solid-state-like density which is expected when accelerating in the regime of radiation pressure acceleration (RPA)^9–12^. The densities of ion bunches delivered by conventional accelerators are orders of magnitude lower and thus insufficient to allow for the realization of the fission-fusion scheme to generate these isotopes.

Besides the still-to-be-shown acceleration of heavy ions in the RPA regime, the fission-fusion reaction process requires kinetic energies of the laser-accelerated heavy ions around 7 MeV/u (for $^{232}$Th) to exceed the fission barrier of the heavy ions. In this paper, we present for the first time experimental energy spectra from laser-accelerated heavy (gold) ions with kinetic energies that exceed this threshold of 7 MeV/u, denoting a first milestone towards the realization of this reaction mechanism. Furthermore, we show measured gold ion charge-state distributions with an unprecedented, individual-charge-state resolution. This data reveals a remarkable target thickness dependency, which lacks a straight-forward explanation by established ionization models and calls for further theoretical studies.

## Experimental setup

**Figure 1:**
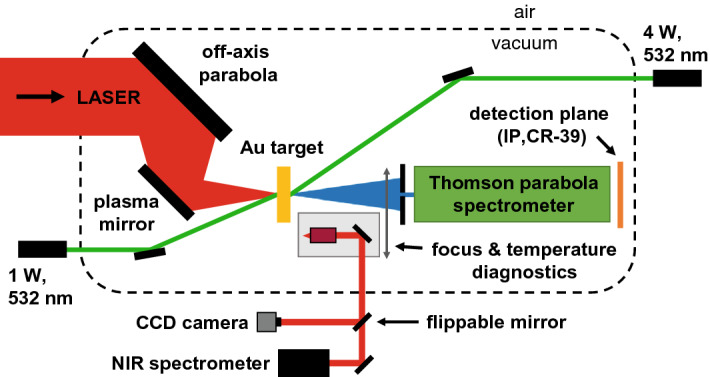
Experimental setup at the PHELIX laser.

The experiment was conducted at the PHELIX laser of the GSI Helmholtzzentrum für Schwerionenforschung in Darmstadt, Germany^13^. A schematic overview of the experimental setup is provided in Fig. 1. The PHELIX laser delivered pulses with energies of $185 \pm 15$ J and durations of 500 fs at a central wavelength of 1054 nm. The beam was focused by an f/1.6 off-axis parabolic mirror to an area of about $14.5 \pm 0.5$ $\upmu$m$^2$, which contained around 23 % of the laser energy. UV fused silica windows were used as single inline plasma mirrors for contrast enhancement. The cycle-averaged peak intensity is estimated to be $\left( 4.1\pm 0.9\right) \, \times \,10^{20}$ $\text {W}\,\text {cm}^{-2}$.

Gold targets with thicknesses of 25, 45, 100, 300 and 500 nm ($\pm 10\,$%) were provided by the LMU and GSI target laboratories. Surface contaminants were removed by radiative heating via two cw lasers at a wavelength of 532 nm (variable laser output power up to 4 W at the target front side, up to 1 W at the rear side). The thermal spectrum has been recorded outside the vacuum chamber via the optical path of the focus diagnostics by an NIR spectrometer. Although a reliable real-time determination of the foil surface temperature from the thermal spectra was not feasible during the beamtime, the targets were heated as close as possible to their melting point (1064$\,^\circ$C) by carefully increasing the laser power, while watching the thermal spectrum and the target foil surface.

A Thomson parabola spectrometer (TPS) was optimized for heavy ion characterization. The TPS features a magnetic dipole field with an average design field strength of about 850 mT along a distance of 168 mm. An electric field of up to 30 kV/cm can be applied along a distance of up to 570 mm. The TPS was equipped with a stainless-steel entrance pinhole of 100 $\upmu$m diameter and a thickness of 50 $\upmu$m (sufficient to stop gold ions with energies up to about 12 MeV/u^14^), collecting the ions emitted in a solid angle of $1.5\,\times \,10^{-5}$ msr in target normal direction. CR-39 sheets with dimensions of $200 \times 95 \times 1$ mm$^3$ were employed as passive ion detectors. After irradiation, they were etched in six-molar NaOH for 45 minutes at a temperature of 80$\,^\circ$C and subsequently scanned by a Zeiss Axiotron II autofocus microscope using the pattern recognition software SAMAICA^15^. This software automatically detects ion impacts on the CR-39 sheets and fits an ellipse to each identified track, providing its absolute position together with information about the enclosed area (size) of the track, its central brightness (when being perpendicularly illuminated) and its ellipticity, all used for further data processing.

**Figure 2:**
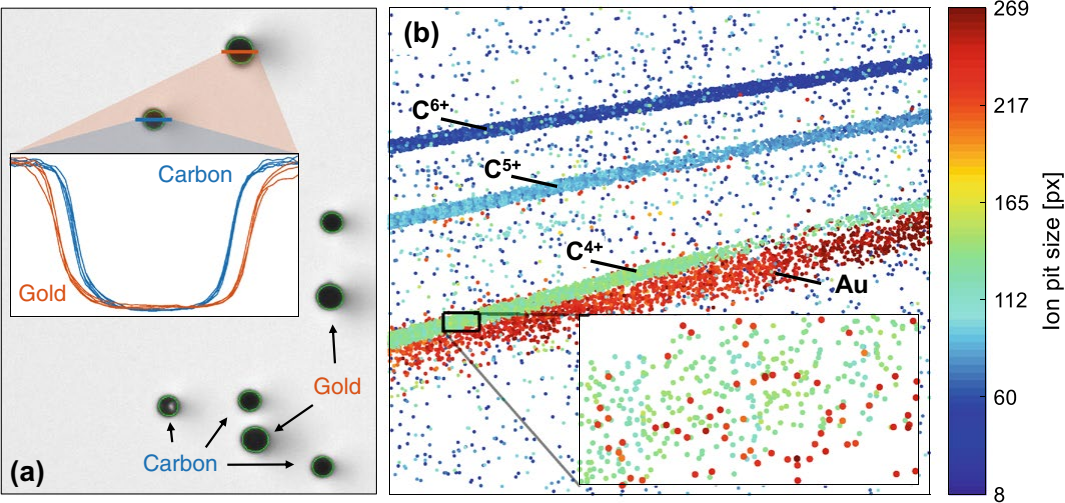
(**a**) Microscope image of an irradiated CR-39 sheet. The black dots originate from gold and carbon impacts and have been registered by the software SAMAICA, which fitted ellipses around the dots (in green). Carbon and gold ion impacts can be discriminated by their size, which is confirmed by the inset showing lineouts through several pits of the respective ion species. The FWHM of the carbon and gold ion impacts in this specific example are about 32 and 38 pixels, respectively (not shown in the figure). (**b**) Scanned CR-39 image from a detector placed behind the TPS. Each point corresponds to an ion impact. The color map visualizes the ion pit size in form of the enclosed area (number of enclosed pixels). The visible structures can be assigned to C$^{6+}$, C$^{5+}$, C$^{4+}$ and gold ions, respectively. This image shows the dependency of the ion pit size both on ion species (carbon ions in green and gold ions in red can be distinguished even in their overlapping region, see inset) and on the particles’ kinetic energies.

A major advantage of CR-39 is that the dimensions of the impact pits are strongly related to the projectile ions. For example, their ellipticity provides information about the angle of incidence of the impinging ion^16^. Moreover, their size is depending on the ion energy^17^ as well as on the projectile species^18^. Figure 2a exemplarily shows a microscope image with black dots originating from gold and carbon impacts. They have been recognized by SAMAICA, which fitted green ellipses around the dots. The pits originating from heavy gold ions have larger diameters than impacts from the lighter carbon ions, which is confirmed by the inset showing lineouts through several pits from both species (in this specific example, the carbon impact diameter is about 15$\%$ smaller than that of the gold ions). Thus, the two ion species can be distinguished from each other. The relevance of this can be seen from Fig. 2b, which shows a scanned CR-39 image from the detector behind the TPS. The color map refers to the size of the individual ion pits: red pits have large diameters, while blue pits correspond to smaller diameters. In the present case, three curves originating from carbon ions with charge states 4+ to 6+ and an additional gold curve (with no resolvable charge information) are visible. The C$^{4+}$ line in green overlaps with the gold ion pits (in red), which is in more detail shown in the inset. The carbon impacts can still be differentiated from gold ions, which is not possible when employing, for example, an imaging plate or other areal detectors. Furthermore, the dependence of the ion pit sizes on the kinetic energy can be seen from the figure as well. The spectrometer is energy selective and deflects carbon ions with a higher charge state more than carbons with a lower charge state but the same velocity. In case of Fig. 2b, all three carbon lines show different energy ranges. The carbon ions from the C$^{6+}$ (C$^{5+}$) line have higher kinetic energies than the carbon ions from the C$^{5+}$ (C$^{4+}$) curve. As a consequence, they deposit less energy on the CR-39 surface, which generally results in smaller pit diameters, which can be seen from the color code.

**Figure 3:**
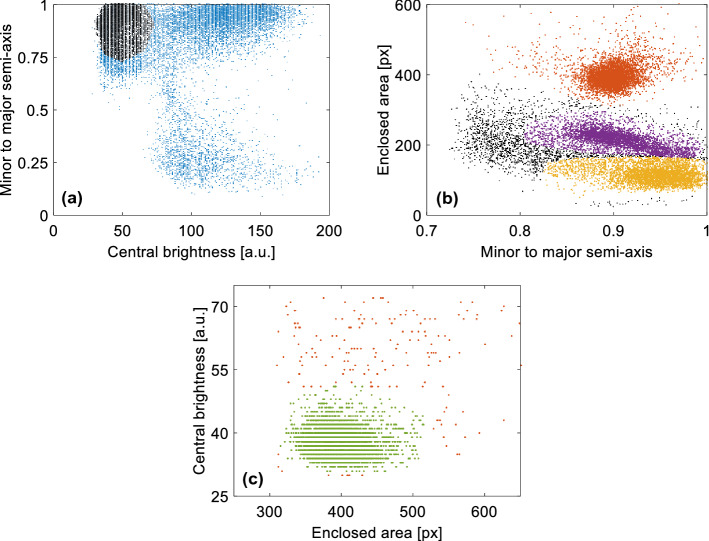
Illustration of the background discrimination using the raw data acquired with CR-39 track detectors. Only pits are accepted, whose ellipse parameters lie within certain boundaries, that can be attributed to ion signal. In total, three filtering stages have been applied to the CR-39 data. The accepted data points in (**a**) are drawn in black. In (**b**), the ion species can be separated by their pit sizes: orange refers to gold ions, violet to oxygens and yellow to carbons. (**c**) The last filtering step for the example of gold ions. The accepted data points are displayed in green. The two-dimensional gate conditions in each step have been defined by hand. The selected regions around the parameter correlations were chosen as small as possible in order to exclude a maximum number of background data points, but large enough that no correlated patterns related to the observed ion curves - especially for heavy ions—appeared in the discarded data.

Selection between the heavy and light ion pits was finally achieved by applying a three-stage filtering process via (conservatively tight chosen) graphical cuts to the 2D distributions of (i) minor-to-major semi axis ratio versus central brightness, (ii) minor-to-major semi axis ratio versus the enclosed area of the fitted ellipse and (iii) central brightness versus enclosed area. The three panels of Fig. 3 illustrate these selection steps for the example of a 500 nm thick, unheated gold foil.

The magnetic field of the TPS was calibrated by two proton energy cutoffs at 19.92 and 22.73 MeV on imaging plates (IPs) placed behind $793\pm 1$ $\upmu$m and $998\pm 1$ $\upmu$m thick copper plates (with an average range straggling of 31 and 37 $\upmu$m, respectively)^14^. Additionally, the energy calibration was confirmed by a carbon ion energy cutoff of 18.9 MeV/u on an IP behind a 1 mm thick CR-39 sheet.

**Figure 4:**
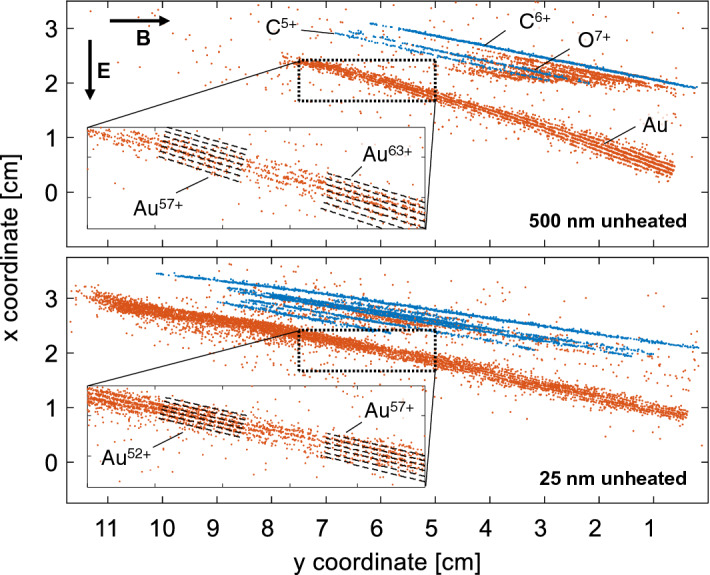
Raw data recorded with CR-39 for shots on unheated foils with a thickness of 500 nm (top), which delivered the best charge-state resolution and 25 nm (bottom), which resulted in the highest gold ion energies. The blue data points correspond to light ions, while the orange ones visualize larger pits caused by heavier ions, in particular gold. The grey dashed lines show the calculated lines for the mass-to-charge ratios of 2, 2.5 and 3, respectively. Details of the gold traces are shown in the insets. As indicated, the magnetic (electric) field deflected the ions to the right (downwards).

Exemplarily, the raw data from two shots on unheated gold foils with thicknesses of 25 nm (which delivered the highest gold ion energies) and 500 nm (with the best gold ion charge-state resolution) are shown in Fig. 4. The ion pits could easily be identified and discriminated on the CR-39 due to their different pit sizes. The blue data points correspond to light ion impacts (carbon and oxygen) and are arranged along thin, curved traces, which can be uniquely assigned to specific mass-to-charge ratios. The orange data points originate from pits on the CR-39 with larger diameters and, thus, were associated with heavy ions, in particular gold. The gold ion impacts are also arranged along thin, curved traces, which lie much closer to each other, as expected for the smaller differences in their mass-to-charge ratio. Detailed views of the gold ion traces in the insets of Fig. 4 confirm the clearly distinguishable gold ion traces (below about 4 MeV/u).

**Figure 5:**
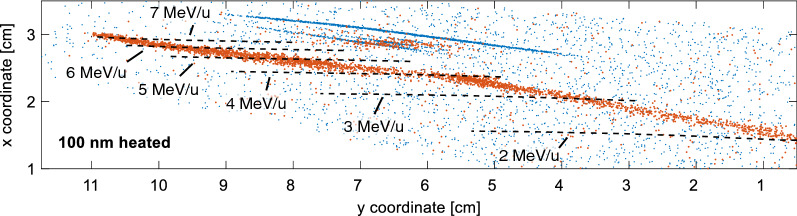
CR-39 raw data after background removal for a laser shot onto a 100 nm thin gold foil that was laser-heated before the shot for surface cleaning. The iso-energy lines starting from 2 MeV/u up to 7 MeV/u are indicated by black dotted lines.

Figure 5 displays the CR-39 raw data after background removal for a shot onto a 100 nm laser-heated gold foil. Iso-energy lines starting from 2 MeV/u up to 7 MeV/u are indicated by black dotted lines. Similarly large ion pits as in the lower energy region are clearly observed, demonstrating that Au ions have been accelerated up to energies around and beyond 7 MeV/u.

We reproducibly observed large pits in the mass-to-charge region between 2 and 2.5 (orange dots below the carbon/oxygen traces). In some shots, their faint occurance was sufficient to reveal individual, closely spaced traces. Both the large pit size and this close spacing suggest rather heavy ions as cause for this signal. Assuming consecutive charge states of a single mass number, we find the possible mass number to be between 70 and 100. It is interesting that fission fragments from a heavy-ion induced fissioning (Au + C) system after particle evaporation would qualify for this region. We note that fission fragments had been identified in PW-laser-plasma experiments^19^ and could in our case also originate from in-target fission caused by energetic carbon^20^ or oxygen^21^ ions, which we also observed regularly in each shot.

## Results and discussion

**Figure 6:**
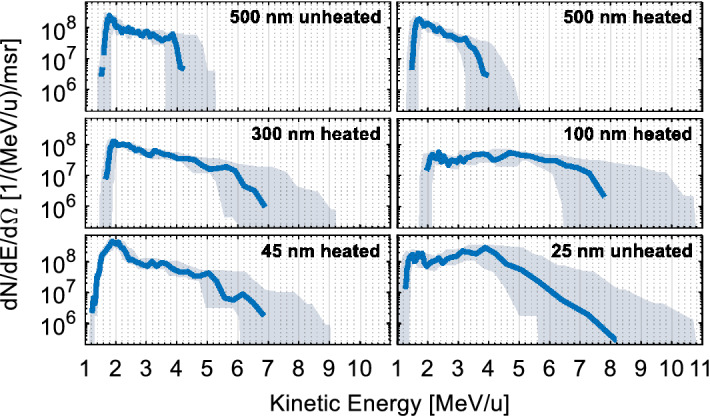
Gold ion energy spectra for shots on gold foils with varying thickness, integrated over all charge states. The grey error margin includes uncertainties due to the calibration of the magnetic field, the determination of the exact charge-state range and due to the intrinsic energy resolution of the TPS. Slight uncertainties of detector positioning and ion numbers have been included as well.

In total, accelerated ions from seven laser shots on gold foils with varying thickness were detected on full CR-39 sheets. Additionally, after an initial learning phase, 15 shots have been detected on slotted CR-39 sheets placed in front of imaging plates, allowing for a fast assessment of the data quality after the laser shot. However, it turned out that these inhomogeneous track detectors could not reliably be analyzed using our automatic autofocus scanning system. Out of the seven shots on full CR-39, one was not suitable for a reliable determination of the gold ion charge states. The remaining six shots have been analyzed and the resulting ion spectra (integrated over all charge states) are shown in Fig. 6. While from the thickest targets (500 nm) gold ions have been accelerated up to kinetic energies around 4 MeV/u, shots on all other foils have reliably delivered maximum gold ion energies above 6 MeV/u, with shot-to-shot fluctuations of about 1 MeV/u as can be seen in Fig. 6. The best results were achieved for the shots on foils with a thickness of 100 nm and 25 nm, where gold ion cutoff kinetic energies around and beyond 7 MeV/u were observed.

**Figure 7:**
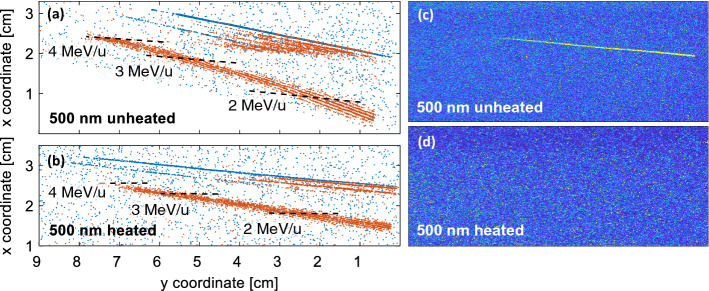
Left: CR-39 raw data after background discrimination for laser shots on (**a**) unheated and (**b**) heated 500 nm thick gold foils. Right: IP raw data for laser shots on (**c**) unheated and (**d**) heated 500 nm gold foils.

The target surface cleaning process with a simultaneous radiative heating of both front and rear surfaces was very effective, as the proton signal was significantly reduced for all shots on heated foils, which was recorded on IPs behind the CR-39 detectors. A direct comparison is only available for the target thickness of 500 nm, for which the CR-39 raw data after background discrimination are shown in Fig. 7a,b. While efficiently removing protons from the target surface (not visible on CR-39, which is transparent to protons in the detectable energy range, but on the IPs, which have been placed behind the CR-39, see Fig. 7c,d), the radiative target cleaning rather (slightly) enhanced the kinetic cutoff energies of light ions (carbon, oxygen). Conversely, the gold bulk species energies remained rather unaffected. In fact, the highest gold ion energies were achieved for a shot on an unheated 25 nm thick gold foil. This is in contrast to former experiments^2,22–24^.

**Figure 8:**
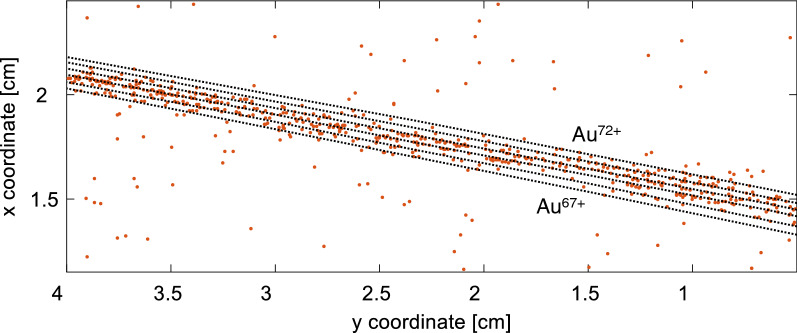
CR-39 raw data with reconstructed curves (dotted lines) in the charge state range of $67^+ - 72^+$ for the assignment of the manually traced data for heavy ion pits.

**Figure 9:**
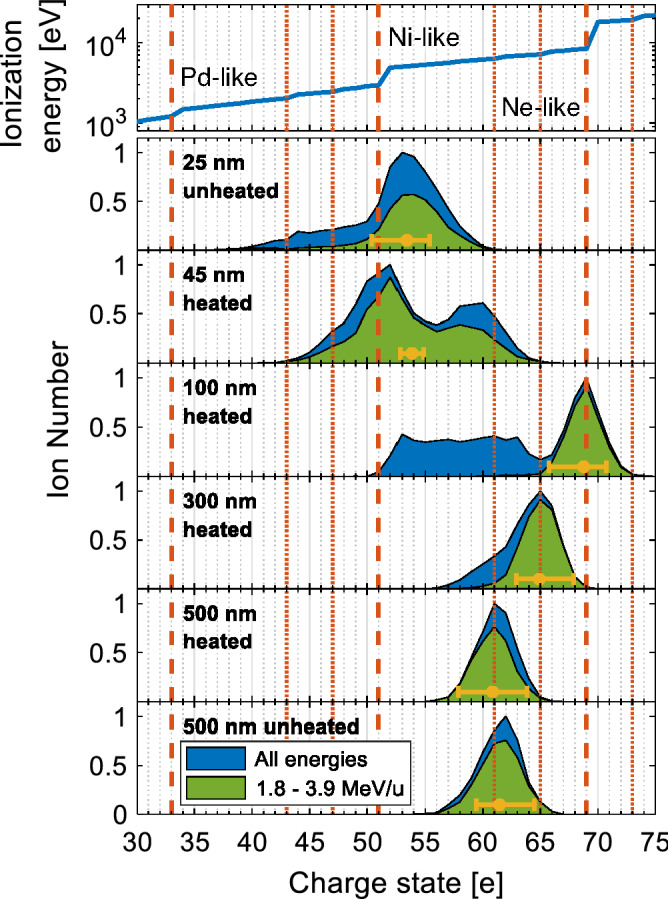
Comparison of the measured gold ion charge states from shots on gold foils with varying thickness to steps in the gold ionization energy indicated by the vertical orange lines (dashed lines correspond to larger, dotted lines to smaller steps). The blue distributions show the gold ion numbers for each charge state integrated over all energies. The green distributions display the number of gold ions integrated between 1.8 and 3.9 MeV/u, for which the individual charge states were resolvable for most of the shots. The yellow point depicts the mean charge state of the green distribution with an error bar showing the uncertainty of the total charge state range. The distributions are normalized to the respective maximum of the blue curves.

Besides these encouraging kinetic energies, we detected the laser-accelerated gold ions with an unprecedented charge-state resolution, which enabled us for the first time to resolve individual charge states up to energies of about 4 MeV/u. The measured gold ion charge-state distributions, normalized to their maximum value, are plotted in Fig. 9, integrated over all energies (blue) and within an energy range between 1.8 and 3.9 MeV/u (green), where individual charge states could be resolved for most of the shots. It is obvious that the width of the charge-state distributions increases towards thinner targets. The TPS allowed measuring charge states higher than $(36^{+2}_{-3})^+$ for a foil thickness of 25 nm, while the highest measured charge state is $(72^{+2}_{-3})^+$ from a 100 nm thick gold foil, which reaches the recently published record ionization value of $72^+$, representing the highest charge state of gold that has been observed with laser acceleration so far^25^. Figure 8 illustrates this highest charge state region by showing the corresponding CR-39 raw data of heavy (gold) ions together with the reconstructed curves for their charge-state assignment.

A significant difference between the charge-state distributions from heated and unheated foils was not observed.

It is striking that each of the charge-state distributions—even the broad ones for thinner target foils—exhibits a clear maximum. Prominent peaks are also visible at individual charge states in the respective distributions shown in the simulation papers^4–6^, with a predominant occupation of the charge state 51$^+$. In reference^6^ gold ion charge-state distributions were simulated, using a multi-dimensional (2D3V) particle-in-cell code, for PHELIX-like laser pulses (500 fs, 60 J, $2.4\,\times \,10^{20}$ $\text {W}\,\text {cm}^{-2}$) impinging onto a 500 nm thick gold foil. The resulting charge-state distribution reveals a sharp cutoff at 51$^+$, which by more than an order of magnitude dominates the distribution. This is in clear discrepancy to our findings of a distribution centered around 61$^+$ for the same target thickness, as shown in the bottom two panels of Fig. 9.

These peaks can be attributed to large steps in the ionization potential, which is shown for the sequential ionization of gold atoms in the topmost panel of Fig. 9^26^. The thick, dashed orange lines mark positions in the charge-state spectra from major steps in the ionization potential that occur for closed atomic shells: 3$3^+$ is palladium-like with a closed 4d shell, 51$^+$ is nickel-like with a closed 3d shell and 69$^+$ is like the noble gas neon with a closed 2p shell. The thinner, dotted orange lines visualize steps in the ionization potential with step sizes that are at least a factor of 2 higher than neighboring steps, which result mostly from closed subshells as well.

However, despite the single-charge-state resolution, the peaks in the measured distributions are not as sharp as in the earlier quoted simulations^4–6^, where distinct peaks stand out compared to the neighboring charge states. Instead, the measured maxima are broadened and distributed over a width of at least three charge states. Nevertheless, these peaks coincide – within their uncertainties for the charge-state range – very well with the positions of the major and minor steps in the ionization potential and show a remarkable thickness dependency: the charge states from thinner foils (< 50 nm) peak around 51$^+$ with a rather broad underlying charge-state distribution, while gold ions from thicker foils are quite closely spread around maxima at much higher charge states. The distributions for the two shots on 500 nm thick gold foils are in excellent agreement with each other and the highest populated charge state lies around 61$^+$ for both cases. For decreasing foil thicknesses, the widths of the charge-state distributions increase and their peak positions move towards higher values, all coinciding with major and minor steps in the ionization energies (around 65$^+$ for 300 nm and 69$^+$ for 100 nm). At a gold foil thickness of 100 nm, a uniform distribution at lower charge states between 51$^+$ and 65$^+$ arises in addition to the peak at 69$^+$. This appears to be the onset of a transition towards the charge-state spectra from thinner foils with thicknesses of 45 and 25 nm, which are relatively broad compared to the thicker foils and located at much lower charge states.

A straight-forward explanation for the measured gold ion charge-state distributions and especially the observed foil thickness dependency could not be found in the framework of established ionization mechanisms (tunnel and electron impact ionization). Regarding the tunnel ionization, the established formulae applying the ADK-model^27,28^ predict for the here measured peak intensity of $\left( 4.1\pm 0.9\right) \,\times \,10^{20}$ $\text {W}\,\text {cm}^{-2}$ the ionization of gold up to 51$^+$, which corresponds to the large step in the ionization potential for a nickel-like configuration. However, the cycle-averaged peak intensity is naturally a factor of two lower than the instantaneous peak value, which acts on the ions in the plasma. Additionally considering the long laser pulse duration, it is possible that the target foil surface expands and becomes relativistically transparent before the laser pulse has ended. In this case, the laser penetrates the foil until it faces deeper target layers with densities above the critical value, at which the laser is reflected back. This would further enhance the intensity by a factor of 4, yielding a peak value of about $3\,\times \,10^{21}$ $\text {W}\,\text {cm}^{-2}$, which would already be sufficient to ionize gold up to 61$^+$ to 65$^+$. The occurrence of relativistic self-focusing is conceivable as well^29^, which would considerably increase the laser intensity and the generation of charge states of 69$^+$ (requiring an intensity of $5\, \times \,10^{21}$ $\text {W}\,\text {cm}^{-2}$) and even above becomes viable.

For the assessment of the relevance of collisional ionization, the Lotz formula^30^ has been used with the ionization potentials from reference^26^ as input instead of the binding energies. Assuming a gold foil thickness of 500 nm, we derive a total electron number of $N = Z\cdot 4.4\times 10^{11}$ and end up with a probability of about $4\,\%$ for the ionization from $51^+$ to $52^+$ and of about $0.6\,\%$ for the ionization from $69^+$ to $70^+$. The influence of a possible return current from regions surrounding the focal spot as discussed in reference^31^, which could potentiate the number of contributing electrons, in particular at low kinetic energies, and thus the ionization probabilities, has not yet been considered.

**Figure 10:**
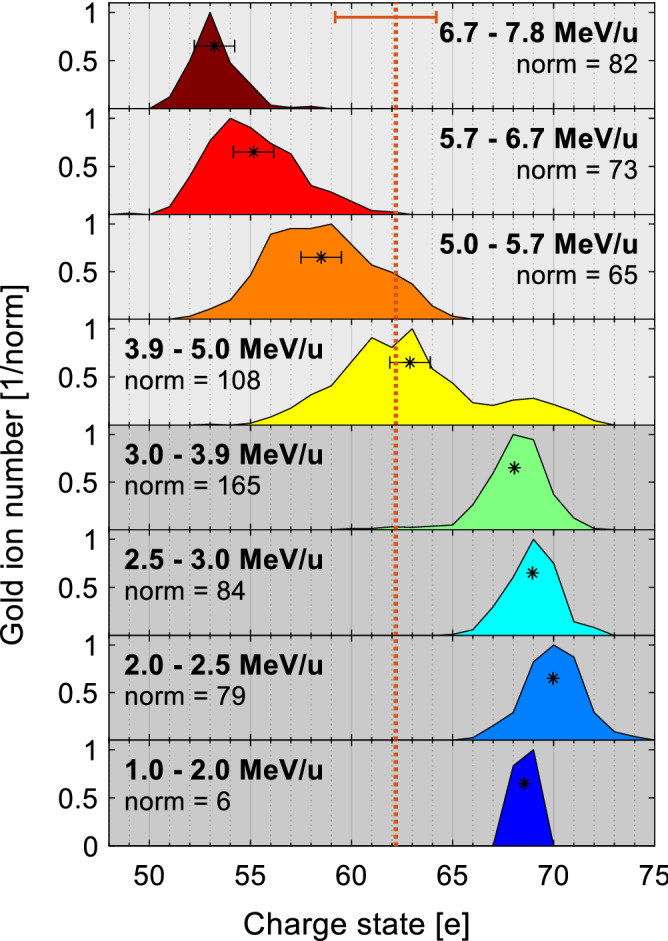
Energy-dependent gold ion charge-state distributions for a shot on a heated, 100 nm thick gold foil. The energies are increasing from the bottom to the top, which is also visualized by the face colors of the distributions (blue means low, red means high energy). The ion numbers of each distribution were normalized to their respective maximum, which is stated for each panel individually (‘norm = X’). The respective mean value has been marked by an asterisk (*) for each charge-state distribution. The error bars in the four topmost panels indicate the charge-state resolution within the shown distribution. The total charge state mean value, averaged over all kinetic energies, is indicated by the dotted, orange line. In the topmost plot, the uncertainty of the charge-state range itself is indicated by the orange error bar.

Figure 10 shows the energy-resolved gold ion charge-state distribution, exemplarily for a shot on a heated, 100 nm thick gold foil. The kinetic gold ion energies are increasing from the bottom towards the top. The respective energy intervals are stated in each panel. It is striking that the gold ion charge states decrease with increasing kinetic energy, especially for energies exceeding 4 MeV/u. At a first glance, this appears counterintuitive, as ions with lower mass-to-charge ratio are generally exposed to higher acceleration forces. The authors of reference^6^ have observed a similar behavior in their simulations. They explained their finding by the sequential nature of the ionization dynamics in combination with the relatively long laser pulse: after an initially similar degree of ionization, faster particles leave the high-ionizing-field area relatively early and thus keep their lower charge state during their acceleration phase, while slower particles remain in the high-field region, being further ionized to higher charge states. Despite their more efficient acceleration, these particles cannot catch up with the lowly charged particles due to the limited remaining acceleration time.

A comparable methodology was used in the recently reported experiment on gold ion acceleration at the CoReLS facility^3^, however, using quite different laser pulse and target properties, as shown in Table 1. Compared to our experimental scenario at the PHELIX laser, in reference^3^ a considerably shorter laser pulse (by a factor of about 23) impinged with much lower pulse energy (ca. 8$\%$ of our case), but about 27 times higher focused intensity on a composite CNF$+$gold foil target (a single gold foil in our case). The featureless gold ion charge-state distribution spanned the region between 37$^+$ and 61$^+$, broadly centered around the charge state 51$^+$. While in reference^3^ comparable maximum values for the gold ion kinetic energies and charge states were observed, a clear difference was observed in the much more pronounced dependence of the gold ion energy as a function of the target heating. While in reference^3^ about a factor of 3 improvement from no heating to 40 s target heating is reported, our data do not feature any clear improvement, most likely due to the much longer laser pulse duration.

**Table 1:** Comparison of experimental parameters of this work, performed at the PHELIX laser, with the experiment at CoReLS from reference^3^.

	This work (PHELIX)	CoReLS^3^
Pulse energy	185(15) J	14–15 J
Pulse length	500 fs	22 fs
Focal spot	14.5(5) $\upmu$m$^2$	2.83 $\upmu$m$^2$
Focused intensity	$4.1(9)\,\times \,10^{20}$ $\text {W}\,\text {cm}^{-2}$	$1.1(4)\,\times \,10^{22}$ $\text {W}\,\text {cm}^{-2}$
Target	25–500 nm Au foil	60 $\upmu$m CNF + 150 nm Au foil
Au charge states	$\le 72(+2\,\,\, -3)^+$	$\le 61^+$
Au kinetic energy	< 1.5 GeV	< 1.1 GeV

In conclusion, we have detected laser-accelerated heavy (gold) ions with energies exceeding 7 MeV/u, which represents the demonstration of an important milestone towards the realization of the novel fission-fusion reaction mechanism. Due to the low relative mass difference, the dynamics of the laser-based acceleration of gold ions is expected to behave in a similar manner as even heavier ion species as, e.g., thorium with a mass number of 232, which was proposed as promising target material for the fission-fusion reaction mechanism by Habs et al.^7^. The transit from gold to thorium as target material is the next step in the preparation of this reaction process, for which the target cleaning will gain in importance regarding the high degree of oxidation on thorium surfaces.

The density of the gold ion bunches can be estimated based on the absolute particle numbers from our analysis, assuming the (non-relativistic) mean velocity to be $v=c\sqrt{\frac{2E_u}{m_pc^2}}$ with $E_u$ being the kinetic energy of the ions per nucleon, $m_p$ the proton rest mass and *c* the vacuum speed of light. With the time of flight of $t = l/v$ and a dispersion of $\Delta t = \frac{l\Delta v}{v^2}$, the density can be expressed as

1 $$\begin{aligned} n = \frac{\Delta N(E_u)}{\Delta A \cdot v\Delta t} = \frac{\Delta N(E_u)}{l^2\Delta \Omega \cdot vl\frac{\Delta v}{v^2}} = \frac{2E_u}{l^3}\frac{\Delta N(E_u)}{\Delta \Omega \cdot \Delta E_u} = \frac{2E_u}{l^3}\frac{\Delta N(E_u)}{\Delta \Omega \cdot (0.01 \cdot E_u)}, \end{aligned}$$

with $\Delta N(E_u)$ being the number of gold ions at the energy $E_u$ traversing an area $\Delta A$ at the distance *l* to the target and $\Delta \Omega$ being the solid angle into which the gold ions are emitted. With﻿ typical values of $\frac{\Delta N(E_u)}{\Delta \Omega \cdot (0.01 \cdot E_u)} \approx \frac{10^8}{sr\cdot 0.01E_u}$, the ion bunch density can be approximated with

2 $$\begin{aligned} n(l) = \frac{2\times 10^{10}}{l^3}, \end{aligned}$$

leading at this energy to an ion bunch density around $10^{13}\,\frac{1}{\text {cm}^3}$ ($10^{16}\, \frac{1}{\text {cm}^3}$) at a distance of 1 mm (100 $\upmu$m) from the target. These promising high densities motivate further studies on potential collective effects on the ion stopping or energy loss behavior in solid or gaseous media at even higher laser intensities.

The high charge state resolution in our experiments provide novel, experimental data which challenges theoretical models that rely on the two most commonly used ionization models. Although these processes have the potential to ionize gold atoms up to the measured charge states, neither the remarkable target foil thickness dependency nor the narrow width of the charge-state distributions for thicker gold foils can be explained in a straight-forward and intuitive way. Therefore, this data constitutes a valuable input for further theoretical investigations targeting to the analysis of the exact contributions of different ionization mechanisms in laser-generated plasmas. In the near future similar measurements will also be performed at the CALA in Garching^32^, where short laser pulses (25–27 fs) with pulse energies up to 60 J will be available. In order to gain more insight into the dynamics of the acceleration and ionization mechanisms, spectral and temporal diagnostics of the transmitted light pulses might help to complement the gold ion characterization. Moreover, special attention will be paid to a confirmation and identification of the heavy ion component observed in the region around m/q$=$ 2–2.5.
